# Modeling and Compensation Methods for Trajectory Errors in Continuous Fiber-Reinforced Thermoplastic Composites Using 3D Printing

**DOI:** 10.3390/polym17131865

**Published:** 2025-07-03

**Authors:** Manxian Liu, Sheng Qu, Shuo Li, Xiaoqiang Yan, Wei Li, Yesong Wang

**Affiliations:** 1 School of Mechanical Engineering, University of Science and Technology Beijing, Beijing 100083, China; d202210291@xs.ustb.edu.cn (M.L.); qhosen@live.com (S.Q.); flipped1491@163.com (S.L.); yanxq@ustb.edu.cn (X.Y.); 2 School of Mechanical Engineering, Jiangsu University of Science and Technology, Zhenjiang 212000, China; 3 School of Aerospace Engineering and Applied Mechanics, Tongji University, Shanghai 200092, China

**Keywords:** continuous fiber prepreg filament, 3D printing, trajectory error modeling, trajectory error compensation methods, process parameters

## Abstract

Defects arising from the 3D printing process of continuous fiber-reinforced thermoplastic composites primarily hinder their overall performance. These defects particularly include twisting, folding, and breakage of the fiber bundle, which are induced by printing trajectory errors. This study presents a follow-up theory assumption to address such issues, elucidates the formation mechanism of printing trajectory errors, and examines the impact of key geometric parameters—trace curvature, nozzle diameter, and fiber bundle diameter—on these errors. An error model for printing trajectory is established, accompanied by the proposal of a trajectory error compensation method premised on maximum printable curvature. The presented case study uses CCFRF/PA as an exemplar; here, the printing layer height is 0.1~0.3 mm, the fiber bundle radius is 0.2 mm, and the printing speed is 600 mm/min. The maximum printing curvature, gauged by the printing trajectory of a clothoid, is found to be 0.416 mm^−1^. Experimental results demonstrate that the error model provides accurate predictions of the printed trajectory error, particularly when the printed trajectory forms an obtuse angle. The average prediction deviations for line profile, deviation kurtosis, and deviation area ratio are 36.029%, 47.238%, and 2.045%, respectively. The error compensation effectively mitigates the defects of fiber bundle folding and twisting, while maintaining the printing trajectory error within minimal range. These results indicate that the proposed method substantially enhances the internal defects of 3D printed components and may potentially be applied to other continuous fiber printing types.

## 1. Introduction

In light of the rapid advancements in high-end equipment, there is an escalating demand for materials and structures that exhibit high performance, lightweight characteristics, integration, and multifunctionality. Traditional composite material molding techniques often fall short of these stringent requirements, particularly when fabricating intricate geometric composite structures. Continuous fiber-reinforced thermoplastic composites via 3D printing (CFRTPCs 3DP), a groundbreaking composite material molding technology, offers a streamlined process with high material utilization [1,2,3]. It eliminates the need for molds and facilitates the integrated molding of complex composite structures. This presents a compelling technical solution for the cost-effective and expedited production of lightweight, high-strength composite material structures [4,5,6,7,8,9,10,11,12].

**Figure 1:**
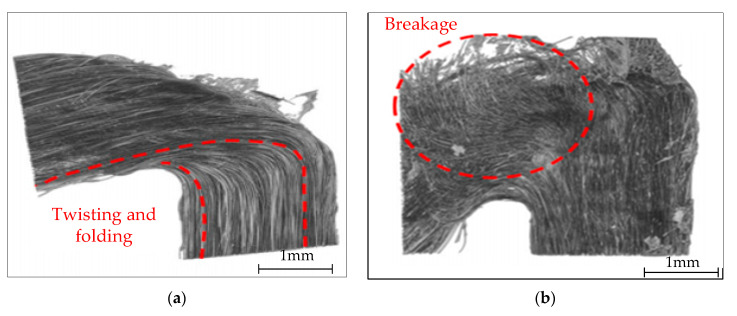
(**a**) Twisted and folded fiber bundles; (**b**) broken fiber bundles.

The current state of CFRTPCs 3DP technology is still in its nascent stage. The strength of printed parts lags significantly behind that of traditional hot-pressed composite parts, with the finished components exhibiting internal defects and insufficient strength [13,14,15,16,17]. This falls short of meeting the mechanical performance requirements of high-end equipment structures. A major issue stems from the multi-source nature and uncertainty of the printing process parameters of continuous fiber prepreg filament (CFPF). This leads to deviations between the actual printing trajectory of CFPF and the designed trajectory during the printing process. These discrepancies are particularly pronounced when printing the bending part, where they can result in distortion, folding, or even fracture of the fiber bundle, as shown in Figure 1. These defects seriously degrade product quality and impede the application and wider adoption of continuous fiber-reinforced composite materials [18].

A multitude of studies indicate that the current CFRTPCs 3DP technology faces significant challenges when fabricating continuous fiber-reinforced composites with intricate shapes and good mechanical properties. The primary determinant affecting the strength, geometric precision, and accuracy of printed components is the printing trajectory [19]. Shiratori et al. [20] examined the impact of printing trajectory process parameters on the performance and defects of printed components. They found that when printing bending trajectories with CFPF, the fiber bundle may twist, fold, or even break. As the radius of the CFPF increases, a larger curvature radius of the fiber trajectory in the bending section is required, leading to greater trajectory errors [21]. Wang et al. [22] discovered that as the corner angle decreases, the printing trajectory transitions at the corner along an approximate arc curve, causing the enclosed gap area between the ideal and printed trajectories to expand. This approximate transition arc has a minimum radius, and the curvature radius of the printed trajectory at the corner must exceed this value; otherwise, it cannot be printed. Zhang et al. [23] investigated the misalignment of fibers during the printing of straight trajectories and the correlation between printing parameters and fiber corner radius. Particularly when using CFPF to print curves, fluctuations, twists, and peeling can occur during the CFPF printing process, similar to automatic fiber placement [24], detrimentally affecting the quality of the printed test pieces. Research has demonstrated that as the diameter of the CFPF and the printing thickness increase, the ability to control accuracy and adhesion performance diminishes [25]. During the printing of any trajectory other than a straight line, the actual printing trajectory of CFPF often deviates from the planned trajectory. These discrepancies become more noticeable in regions with greater curvature and larger fiber bundles. Shang et al. [26] discovered that the resin material did not solidify promptly during the printing process. Consequently, continuous fibers were dragged a certain distance at the peak and valley, resulting in the final printed sine path’s amplitude being less than the design value. Therefore, the difference between the actual amplitude and the design amplitude must be factored into calculations. Kim et al. [27] employed finite element model analysis in the laying of CFPF to minimize defects caused by fiber twisting during the turning process. Wang [28] was the first to propose a mathematical model for the formation of CFPF printing trajectory errors. He investigated the intrinsic relationship between CFPF printing nozzle diameter, minimum curvature radius, trajectory corner angle, and trajectory error through numerical simulation. This work optimized the CFPF printing trajectory and reduced printing trajectory errors. However, despite the detailed exploration of the trajectory error formation mechanism and the examination of their patterns, Wang did not produce a comprehensive theoretical model. Consequently, the scope for compensating printing trajectory errors remains limited. Hao et al. [29] used linear, polynomial spline curves, and flow field functions to parameterize the traction trajectory directly for describing curved fiber trajectories. Niu et al. [30] proposed a method to control the curvature of the reference trajectory via a local curvature correction algorithm. This significantly reduced gap errors such as bending and wrinkling between adjacent trajectories, decreasing the gap error rate from 45.8% to 4.2%. While this trajectory planning approach significantly reduces the gap rate, it overlooks the underlying mechanisms causing trajectory errors. Consequently, it is challenging to improve internal defects. Karimi et al. [31] provide a comprehensive examination of two main FDM mechanisms: in situ fusion and ex situ prepreg, supplementing their discussion with relevant examples using various reinforcing elements. Moreover, they also explore several less commonly used mechanisms. Each mechanism presents its unique set of advantages and disadvantages, suggesting that further refinements and modifications are necessary to enhance the strength of 3D-printed FDM parts, making them comparable to those manufactured using traditional methods. For example, compaction rollers can be attached to the FDM machine to apply more pressure on the printed layers, thereby filling the voids and gaps and leading to improved mechanical properties. Zhang et al. [32] introduced on the three distinct groups of defects that may occur during the additive manufacturing process. And analysis was conducted to summarize the failure behaviors of CFR3DP and to pinpoint the primary causes of such failures.

In summary, existing research has examined the correlation between printing trajectory process parameters and molding defects. While several scholars have explored fiber trajectory errors in the CFPF printing process to varying extents, a comprehensive analysis of the underlying causes of these trajectory defects and errors remains lacking. Notably, the precise relationship between multi-source parameters (such as process, material, and structural parameters) and trajectory error formation has yet to be thoroughly investigated. Such errors significantly influence the size accuracy and internal defects of printed components and can potentially result in printing failures. Consequently, there is an urgent need for further research into the mechanisms of trajectory error formation in CFRTPCs 3DP, as well as trajectory error modeling and compensation techniques.

This study addresses the aforementioned issues by proposing a follow-up theory assumption which elucidates the formation mechanism of trajectory errors in CFRTPCs 3DP. It examines the impact of key geometric parameters, including trajectory curvature, printing nozzle inner diameter, and CFPF diameter, on these trajectory errors. A model for these errors has been developed, and a compensation method, based on maximum printing curvature, is proposed. Additionally, a technique for measuring the maximum printing curvature, based on the printing trajectory of a clothoid, is introduced. The accuracy of the error model and the feasibility of the compensation method are confirmed through error compensation experiments.

## 2. Theory and Method

### 2.1. Assumption of Follow-Up Theory

**Figure 2:**
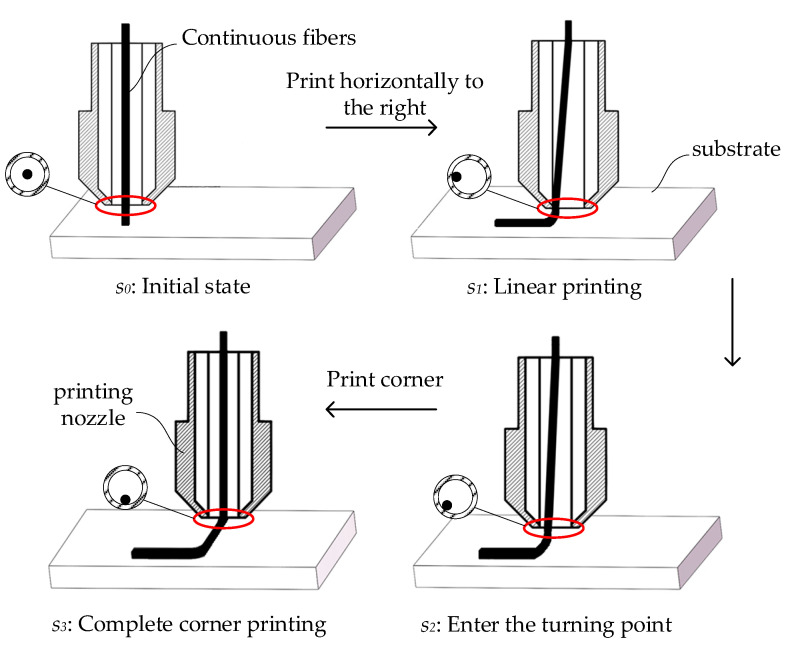
The state of CFPF at the nozzle outlet under different printing trajectories. *s_i_*: State at time point *i*.

As illustrated in Figure 2, the CFRTPCs 3DP trajectory planning typically presumes that the CFPF is cylindrical and coaxial with the printing nozzle. However, this assumption deviates from reality as the extrusion speed of the CFPF surpasses the printing speed, causing an excess of the CFPF to accumulate inside the printing nozzle. Furthermore, the outer layer of resin on the CFPF is often scraped off by the printing nozzle, adding to the material buildup within it. To counter the risk of clogging, the printing nozzle’s inner diameter is designed to be larger than that of the CFPF, allowing for free movement of the CFPF at the nozzle outlet. Consequently, the position of the CFPF at this outlet varies from the ideal printing trajectory during the printing process. As a result, the actual printing trajectory is influenced not only by the diameter of the CFPF, the inner diameter of the printing nozzle, the extrusion speed, and the feed speed, but also by the radius of curvature and the bending angle of the ideal printing trajectory.

This study examines the mechanism underlying printing trajectory errors attributed to specific geometric parameters, namely printing trajectory curvature, CFPF diameter, and printing nozzle inner diameter, premised on the following hypotheses:

1. Disregarding the impacts of adhesion, elasticity, and inertia forces of the CFPF;
2. The impact of the motion precision of 3D printing equipment is disregarded.
3. Within the nozzle, the CFPF is passively extruded and subjected to tension.

**Figure 3:**
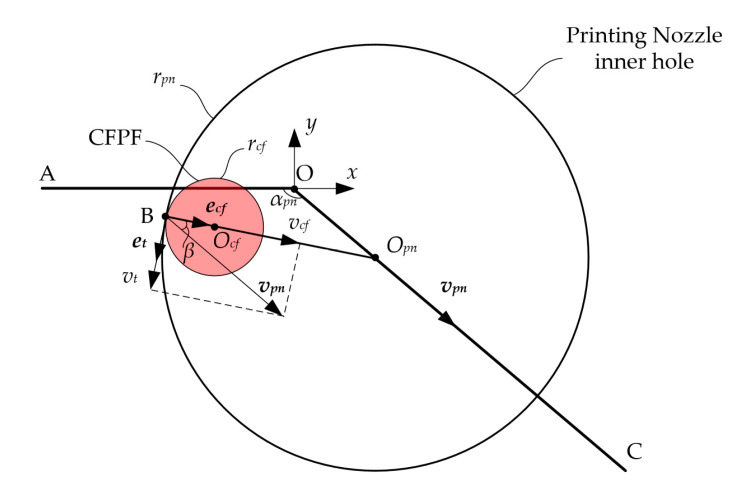
Analysis of CFPF motion, predicated on the assumption of follow-up theory.

Given the aforementioned assumptions, and confined by the physical parameters of the nozzle’s arc-shaped inner wall and the printing feed direction, the CFPF invariably makes contact with the nozzle’s inner wall at a point, where they are tangent to each other. The CFPF moves along the normal speed component of the contact point, i.e., the CFPF generates follow-up movement under the nozzle’s traction, as shown in Figure 3.

Currently, the distance at the center between the CFPF and the nozzle equates to the differential in their radii.

(1) $$\bar{O_{pn}O_{cf}}=\Delta r=r_{pn}-r_{cf}$$

where $r_{cf}$ represents the radius of the CFPF, $r_{pn}$ denotes the radius of the inner wall of the printing nozzle, and Δr is the difference in radius between the CFPF and the nozzle. As per the velocity synthesis theorem in kinematics, when the angle between the normal vector at the point of contact between the CFPF and the inner wall of the nozzle aligns with the feed speed $v_{pn}$ of the nozzle at a certain angle β, the relationship can be expressed as:

(2) $$v_{pn}=v_{t}+v_{cf}=v_{t}e_{t}+v_{cf}e_{cf}$$

(3) $$v_{cf}=\left\|v_{pn}\right\|\cos\beta$$

### 2.2. Mechanism of Printed Trajectory Error Formation

**Figure 4:**
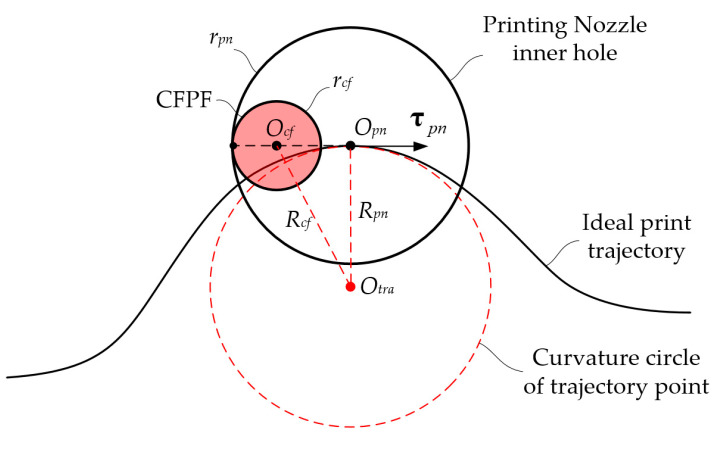
Continuous smooth printing trajectory error formation mechanism analysis.

As shown in Figure 4, when the ideal printing trajectory is a smooth curve, the CFPF exhibits a lag relative to the ideal trajectory point according to the assumption of follow-up theory. It is tangential to the inner wall of the nozzle with its tangent vector running perpendicular to the tangent vector of the printing trajectory point. Notably, the actual printing trajectory is located on the exterior of the ideal trajectory. The parametric equation of the ideal printing trajectory is as follows:

(4) $$\left\{\begin{array}{l} x_{Opn}=\varphi_{pn}\left(t\right)\\ y_{Opn}=\omega_{pn}\left(t\right) \end{array}\right.t:t_{0}\to t_{1}\left(t_{0}\leq t_{1}\right)$$

The radius of curvature for the ideal printing trajectory is as follows:

(5) $$R_{pn}=\frac{{\left[\varphi_{pn}^{\prime 2}\left(t\right)+\omega_{pn}^{\prime 2}\left(t\right)\right]}^{\frac{3}{2}}}{\left|\varphi_{pn}^{\prime }\left(t\right)\omega_{pn}^{\prime \prime }\left(t\right)-\varphi_{pn}^{\prime \prime }\left(t\right)\omega_{pn}^{\prime }\left(t\right)\right|}$$

The tangent vector of the ideal printing trajectory is as follows:

(6) $$\tau_{pn}={\left[\begin{array}{cc} {\varphi^{\prime }}_{pn}\left(t\right) & {\omega^{\prime }}_{pn}\left(t\right) \end{array}\right]}^{T}$$

The unit tangent vector of the ideal printing trajectory is as follows:

(7) $${\hat{\tau }}_{pn}=\frac{\tau_{pn}}{\left\|\tau_{pn}\right\|}$$

The parametric equation of the actual printing trajectory of CFPF is:

(8) $$\left\{\begin{array}{l} x_{Ocf}=\varphi_{cf}\left(t\right)\\ y_{Ocf}=\omega_{cf}\left(t\right) \end{array}\right.t:t_{0}\to t_{1}\left(t_{0}\leq t_{1}\right)$$

According to the assumption of follow-up theory, the actual and ideal printing trajectory points share the same tangent simultaneously, as illustrated in Figure 4:

(9) $$\left[\begin{array}{c} x_{Ocf}\\ y_{Ocf} \end{array}\right]=\left[\begin{array}{c} x_{Opn}\\ y_{Opn} \end{array}\right]-\Delta r{\hat{\tau }}_{pn}$$

The parameterized equation representing the actual printing trajectory of CFPF is determined as:

(10) $$\left\{\begin{array}{l} x_{Ocf}=\varphi_{pn}\left(t\right)-\frac{\Delta r\varphi_{pn}^{\prime }\left(t\right)}{\sqrt{\omega_{pn}^{\prime 2}\left(t\right)+\varphi_{pn}^{\prime 2}\left(t\right)}}\\ y_{Ocf}=\omega_{pn}\left(t\right)-\frac{\Delta r\omega_{pn}^{\prime }\left(t\right)}{\sqrt{\omega_{pn}^{\prime 2}\left(t\right)+\varphi_{pn}^{\prime 2}\left(t\right)}} \end{array}\right.$$

The explicit equations governing the trajectory error model is determined as:

(11) $$\left\{\begin{array}{l} \Delta x=-\frac{\Delta r}{\sqrt{{f^{\prime }}^{2}(x)+1}}\\ \Delta y=-\frac{\Delta rf^{\prime }(x)}{\sqrt{{f^{\prime }}^{2}(x)+1}} \end{array}\right.$$

where y=f(x) is the equation of the ideal printing trajectory.

As depicted in Figure 4, the curvature radius of the actual printing trajectory is:

(12) $$R_{cf}=\sqrt{R_{pn}^{2}+\Delta r^{2}}$$

The discrepancy between the actual and ideal printing trajectory curvature radii is denoted as:

(13) $$\Delta R=\sqrt{R_{pn}^{2}+\Delta r^{2}}-R_{pn}$$

By differentiating the aforementioned equation with respect to ΔR, we can deduce that:

(14) $$\Delta R^{\prime }=\frac{R_{pn}}{\sqrt{R_{pn}^{2}+\Delta r^{2}}}-1\leq 0$$

**Figure 5:**
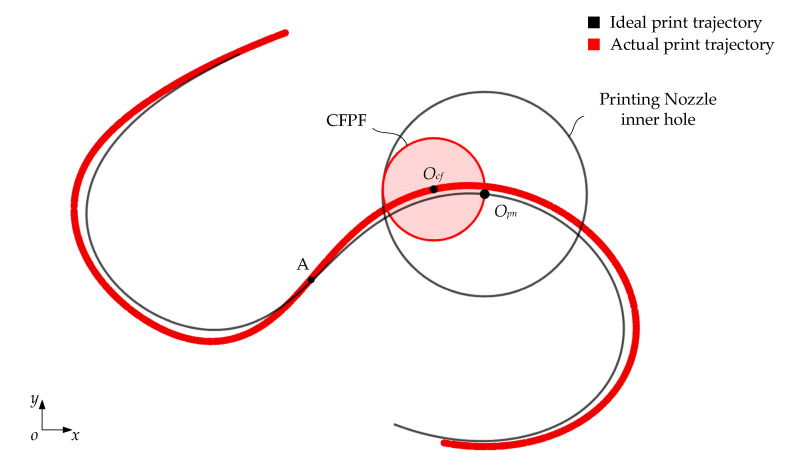
Simulation results of S-shaped continuous smooth 3D printing trajectory.

It is established that Equation (14) is decreasing, implying that an increase in the ideal printing trajectory curvature radius leads to a decrease in the error of the actual printing trajectory. Taking the S-shaped printing trajectory as an example, as shown in Figure 5, it can be seen that:

1. The dragging effect of the nozzle’s inner wall on the CFPF results in a lag at the actual printing point. This subsequently leads to deviations in both the shape and position of the S-shaped printing trajectory.
2. The greater the curvature of the ideal printing trajectory, the greater the printing trajectory error; at the inflection point A where the curvature is zero, the ideal printing trajectory coincides with the actual printing trajectory.
3. As the curvature of the actual printing trajectory decreases, the printed trajectory elongates, and consequently, the envelope area of the printed trajectory expands.

### 2.3. Modeling the Error in Printing Trajectory

The printed trajectory’s geometric errors are characterized by three metrics: line profile, deviation kurtosis, and deviation area ratio. These metrics are defined as follows:

- Line profile

In the simply connected region bounded by the actual and ideal printing trajectories, the actual printing trajectory is situated between two envelope lines. These lines encompass a series of circles with radius ς, whose centers align with the ideal printing trajectory. When ς assumes its minimum value, it represents the line profile of the actual printing trajectory.

- Deviation kurtosis

The ratio of the line profile to the length of the ideal printing trajectory, within the simply connected region bounded by the actual and ideal printing trajectories, is referred to as the deviation kurtosis.

(15) $$\chi =\frac{\varsigma }{L_{pn}}\times 100\%$$

where $L_{pn}$ represents the length of the ideal printing trajectory within this simply connected region, $\chi \in \left[0,+\infty \right)$.

- Deviation area ratio

**Figure 6:**
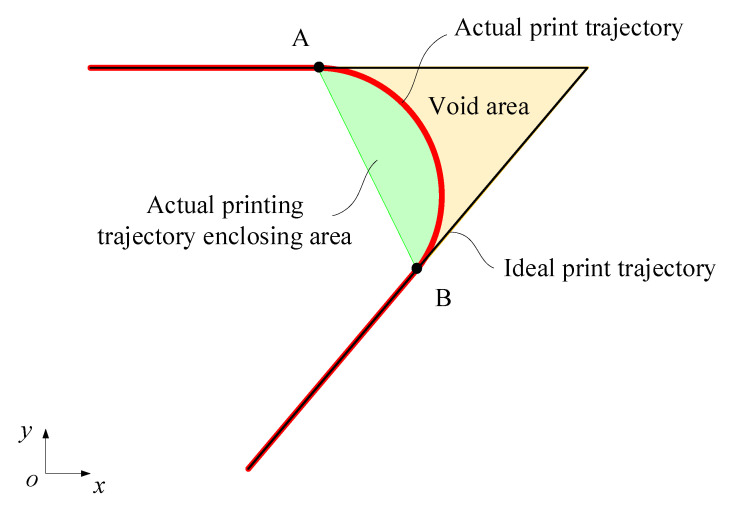
Schematic diagram of printing trajectories error.

The deviation area ratio refers to the proportion of the area enclosed by the actual printing trajectory and the ideal printing trajectory, compared to the area enclosed solely by the ideal printing trajectory.

(16) $$\gamma =\left\{\begin{array}{ll} 0 & s.t.\;S_{pn}=0\\ \frac{\left|S_{cf}-S_{pn}\right|}{S_{pn}}\times 100\% & s.t.\;S_{pn}\neq 0 \end{array}\right.$$

where $S_{pn}$ represents the area bounded by the ideal printing trajectory, while $S_{cf}$ denotes the area encompassed by the actual printing trajectory, $\gamma \in \left[0,+\infty \right)$, as illustrated in Figure 6.

### 2.4. Compensation for Printing Trajectory Errors

In instances where the printing trajectory embodies a continuous, non-smooth curve, the CFPF exhibits a marked susceptibility to distortion and folding, thereby significantly impacting both the accuracy of the print and the mechanical properties of the resultant parts. To mitigate the errors associated with the printing trajectory, this paper introduces a methodology for fitting the bending angle of said trajectory into a printable maximum curvature arc trajectory.

**Figure 7:**
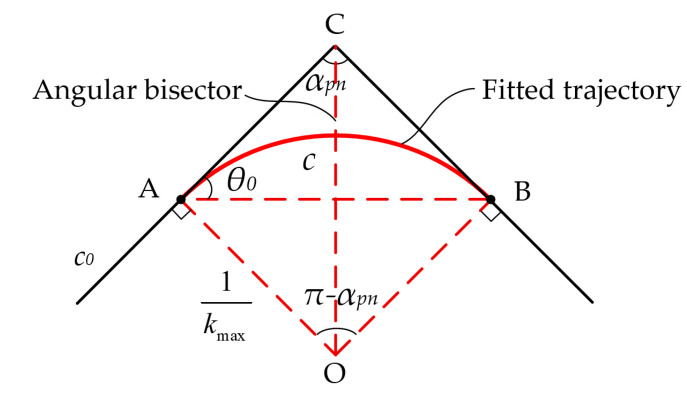
Schematic diagram of trajectory fitting.

As illustrated in Figure 7, the slope of the front bending angle side is denoted as $k_{front}=\tan\theta_{0}$ for the bending angle inflection point $C\left(x_{c},y_{c}\right)$ on the ideal printing trajectory $c_{0}$. By selecting a point O on the bisector of the bending angle of printing trajectory c and drawing a line perpendicular to AC, we have $\bar{OA}=\frac{1}{k_{\max}}$, where $k_{\max}$ represents the maximum curvature that can be printed. In a similar manner, by choosing point B such that OB is perpendicular to BC, we can deduce that:

(17) $$\left[\begin{array}{c} x_{A}\\ y_{A} \end{array}\right]=\left[\begin{array}{c} x_{C}\\ y_{C} \end{array}\right]-\frac{1}{k_{\max}}\cot\frac{\alpha_{pn}}{2}\left[\begin{array}{c} \cos\theta_{0}\\ \sin\theta_{0} \end{array}\right]$$

(18) $$\left[\begin{array}{c} x_{B}\\ y_{B} \end{array}\right]=\left[\begin{array}{c} x_{C}\\ y_{C} \end{array}\right]-\frac{1}{k_{\max}}\cot\frac{\alpha_{pn}}{2}\left[\begin{array}{c} \cos\left(\alpha_{pn}+\theta_{0}\right)\\ \sin\left(\alpha_{pn}+\theta_{0}\right) \end{array}\right]$$

(19) $$\left[\begin{array}{c} x_{O}\\ y_{O} \end{array}\right]=\left[\begin{array}{c} x_{C}\\ y_{C} \end{array}\right]-\frac{1}{k_{\max}}\left[\begin{array}{c} \cos\theta_{0}\cot\frac{\alpha_{pn}}{2}+\sin\theta \\ \sin\theta_{0}\cot\frac{\alpha_{pn}}{2}-\cos\theta_{0} \end{array}\right]$$

where $\alpha_{pn}$ represents the bending angle of the printing trajectory.

As illustrated in Figure 7, an arc is drawn using point A as the origin and point B as the endpoint, based on a radius $\frac{1}{k_{\max}}$ to match the bending angle. The corresponding parametric equation for this fitted arc is:

(20) $$\left[\begin{array}{c} x_{f}\\ y_{f} \end{array}\right]=\left[\begin{array}{c} x_{C}\\ y_{C} \end{array}\right]-\frac{1}{k_{\max}}\left[\begin{array}{c} \cos\theta_{0}\cot\frac{\alpha_{pn}}{2}+\sin\theta_{0}-\cos\phi \\ \sin\theta_{0}\cot\frac{\alpha_{pn}}{2}-\cos\theta_{0}-\sin\phi \end{array}\right]$$

where $\phi \in \left[\theta_{0}-\frac{\pi }{2},\theta_{0}-\alpha_{pn}+\frac{\pi }{2}\right]$. Currently, the arc length of the fitted arc c is as follows:

(21) $$s_{c}=\frac{1}{k_{\max}}\left(\pi -\alpha_{pn}\right)$$

**Figure 8:**
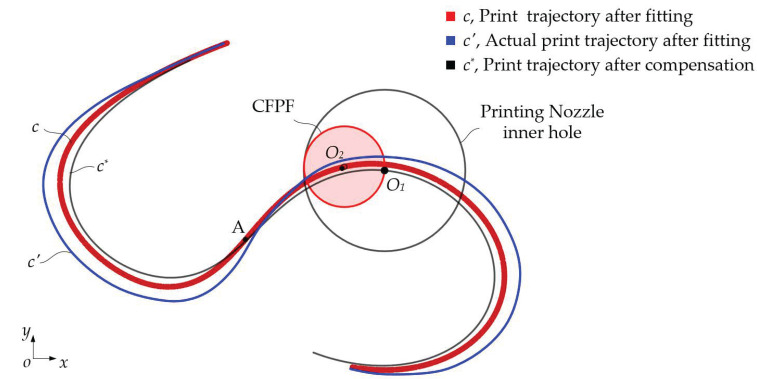
The fitted and compensated printed trajectory.

The analysis of the geometric error mechanism in the printing trajectory reveals that a significant error in the bending angle of the printing trajectory persists after fitting, due to the gap between the CFPF and the printing nozzle, as shown in Figure 8. Consequently, this paper proposes a compensation for the positional error of the fitted printing trajectory using an inverse kinematics model. This approach yields the actual motion trajectory of the printing nozzle while concurrently compensating for the extrusion speed of the CFPF.

Utilizing Equation (9), the parameter equation for the compensated nozzle motion trajectory can be derived by finding the inverse solution for the fitted printed trajectory c.

(22) $$\left[\begin{array}{c} x_{Opn}\\ y_{Opn} \end{array}\right]=\left[\begin{array}{c} x_{Ocf}\\ y_{Ocf} \end{array}\right]+\Delta r{\hat{\tau }}_{pn}$$

(23) $${\hat{\tau }}_{pn}=R_{\theta }{\hat{\tau }}_{cf}$$

(24) $$R_{\theta }=\left[\begin{array}{c} \cos\theta \\ -\sin\theta \end{array}\begin{array}{c} \sin\theta \\ \cos\theta \end{array}\right]=\frac{1}{R_{cf}}\left[\begin{array}{cc} \sqrt{R_{cf}^{2}-\Delta r^{2}} & \Delta r\\ -\Delta r & \sqrt{R_{cf}^{2}-\Delta r^{2}} \end{array}\right]$$

**Figure 9:**
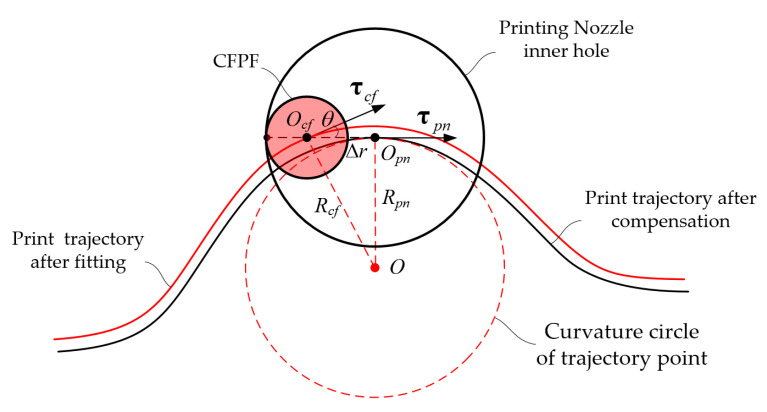
Principle of printing trajectory error compensation.

Upon substituting Equations (23) and (24) into Equation (22), the following is derived:

(25) $$\left[\begin{array}{c} x_{Opn}\\ y_{Opn} \end{array}\right]=\left[\begin{array}{c} x_{Ocf}\\ y_{Ocf} \end{array}\right]+\Delta rk_{cf}\left[\begin{array}{cc} \sqrt{R_{cf}^{2}-\Delta r^{2}} & \Delta r\\ -\Delta r & \sqrt{R_{cf}^{2}-\Delta r^{2}} \end{array}\right]{\hat{\tau }}_{cf}$$

where ${\hat{\tau }}_{cf}$ represents the unit tangent vector of the compensated printing trajectory, ${\hat{\tau }}_{pn}$ signifies the unit tangent vector of the fitted printing trajectory, $R_{cf}$ and $R_{pn}$ are their corresponding curvature radii, $k_{cf}$ is the curvature of the compensated printing trajectory, and $R_{\theta }$ is the rotation transformation matrix from ${\hat{\tau }}_{cf}$ to ${\hat{\tau }}_{pn}$, as depicted in Figure 9.

**Figure 10:**
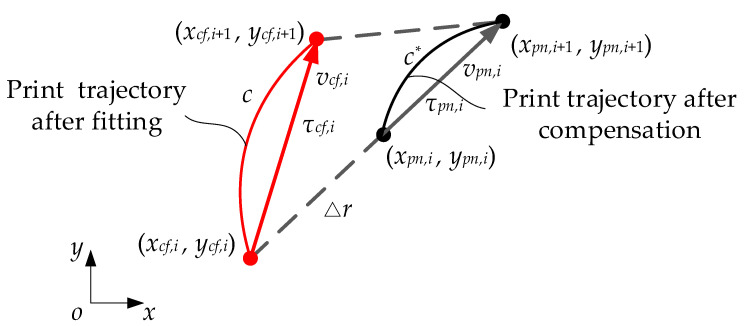
Principle of CFPF extrusion speed compensation.

Subsequently, the interpolation algorithm is utilized to calculate the interpolation points $\left(x_{pn,i},y_{pn,i}\right)$ of the compensated printing trajectory. These points represent the coordinates of the actual motion trajectory of the nozzle. As illustrated in Figure 10, let us assume that the nozzle is moving at a velocity denoted as $v_{pn,i}$; then:

(26) $$t_{pn,i}=\frac{1}{v_{pn,i}}\sqrt{{\left(x_{pn,i+1}-x_{pn,i}\right)}^{2}+{\left(y_{pn,i+1}-y_{pn,i}\right)}^{2}}$$

(27) $$\tau_{pn,i}=\left[\begin{array}{c} x_{pn,i+1}-x_{pn,i}\\ y_{pn,i+1}-y_{pn,i} \end{array}\right]$$

It can be inferred from Equation (10) that:

(28) $$\left[\begin{array}{c} x_{cf,i}\\ y_{cf,i} \end{array}\right]=\left[\begin{array}{c} x_{pn,i}\\ y_{pn,i} \end{array}\right]-\frac{\Delta r}{\Delta l}\left[\begin{array}{c} x_{pn,i+1}-x_{pn,i}\\ y_{pn,i+1}-y_{pn,i} \end{array}\right]$$

(29) $$\Delta l=\sqrt{{\left(x_{pn,i+1}-x_{pn,i}\right)}^{2}+{\left(y_{pn,i+1}-y_{pn,i}\right)}^{2}}$$

Subsequently, the compensated extrusion speed of the CFPF is determined by the equation:

(30) $$v_{cf,i}=\frac{1}{t_{pn,i}}\sqrt{{\left(x_{cf,i+1}-x_{cf,i}\right)}^{2}+{\left(y_{cf,i+1}-y_{cf,i}\right)}^{2}}$$

**Figure 11:**
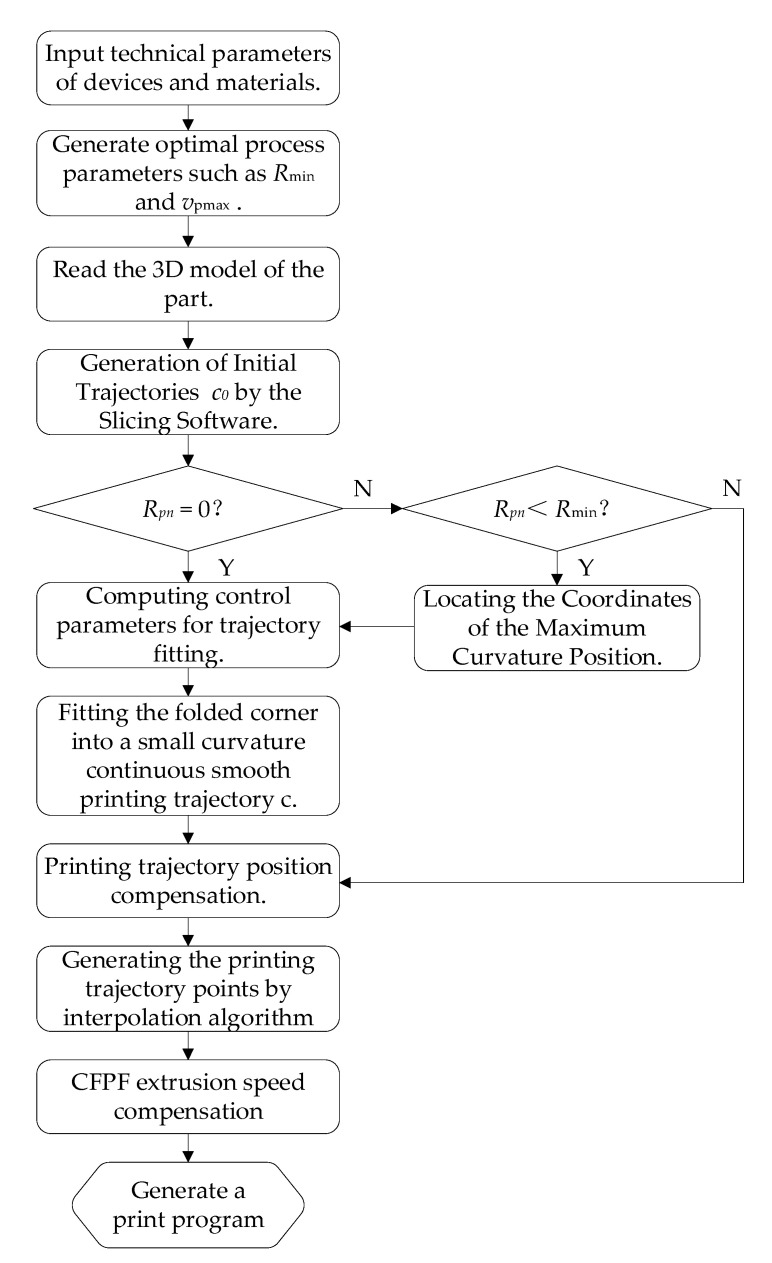
The algorithmic procedure for error compensation of the printing trajectory.

The methodology for compensating printing trajectory errors is illustrated in Figure 11.

## 3. Results and Analysis

### 3.1. Printing Devices Materials and Parameters

**Figure 12:**
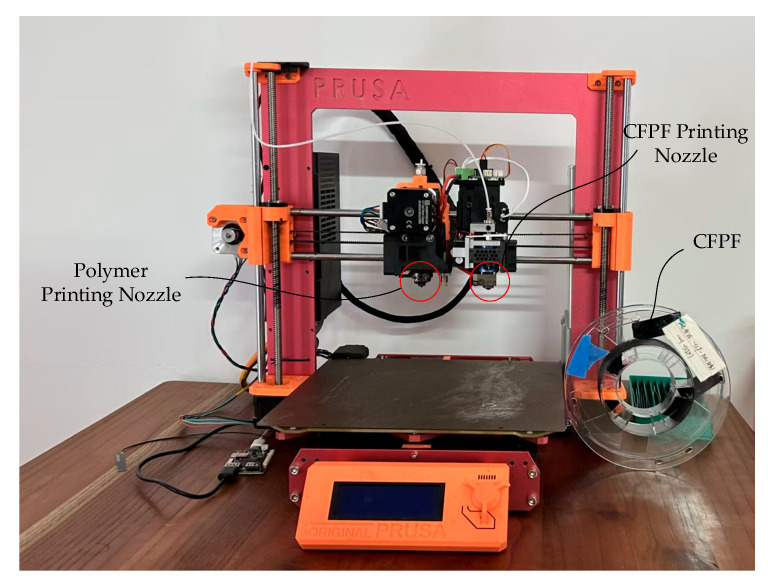
The CFRTPCs 3DP devices.

As illustrated in Figure 12, the Original Prusa MK4S 3D Printer from Prusa Research a.s. was employed for conducting CFPF printing experiments in this study. We designed two independent printing nozzles based on the printer, one of which was used to print CFPF, the other was used to print polymer matrix (PAHT, PLA, ABS, PA, etc.). The diameters of the two printing nozzles are 1.2 mm and 0.4 mm, respectively. Concurrently, to validate the effectiveness of the error compensation model, we employed Matlab R2020a to generate representative trajectories. These were subsequently optimized based on the error compensation model via arc fitting, position compensation, and line interpolation, thereby producing the actual printing trajectory code. The CFPF was pre-prepared prior to printing, and the appropriate nozzle was selected via control command during the actual printing process. This allowed for the separate printing of the matrix and CFPF.

The matrix used in this research is polyamide high temperature (PAHT), which is purchased from the manufacturer FusRock (Suzhou Fosi Luoke New Materials Co., Ltd. Suzhou, China). The continuous fiber-reinforced filament adopted in this study is continuous carbon fiber-reinforced filament (CCFRF), which is also purchased from FusRock. The CFPF utilized in this study is CCFRF/PA, which was prepared using a laboratory-developed device specifically designed for the preparation of CCFRF/PA. Subsequently, the optimal preparation process parameters were determined through rigorous testing. Material parameters and printing parameters are detailed in Table 1 below.

### 3.2. Maximum Printable Curvature Measurement

This study employs the clothoid printing trajectory to determine the maximum printable curvature. It is observed that the curvature *k* increases linearly with the arc length *s*, thus meeting the following condition:

(31) $$k\left(s\right)=c_{k}\cdot s\left(0<c_{k}\right)$$

The variable *c_k_* represents the control parameter for the curvature growth rate. Based on the principles of differential geometry, we can deduce the following conclusions:

(32) $$\theta \left(s\right)=\int_{0}^{s}k\left(t\right)dt=\frac{1}{2}c_{k}s^{2}$$

The variable θ represents the angle between the tangent of the trajectory and the horizontal axis. The clothoid printing trajectory parameter equation can be obtained through Fresnel integration as follows:

(33) $$\left\{\begin{array}{l} x\left(s\right)=\int_{0}^{s}\cos\theta \left(t\right)dt=\int_{0}^{s}\cos\left(\frac{c_{k}}{2}t^{2}\right)dt\\ y\left(s\right)=\int_{0}^{s}\sin\theta \left(t\right)dt=\int_{0}^{s}\sin\left(\frac{c_{k}}{2}t^{2}\right)dt \end{array}\right.$$

**Figure 13:**
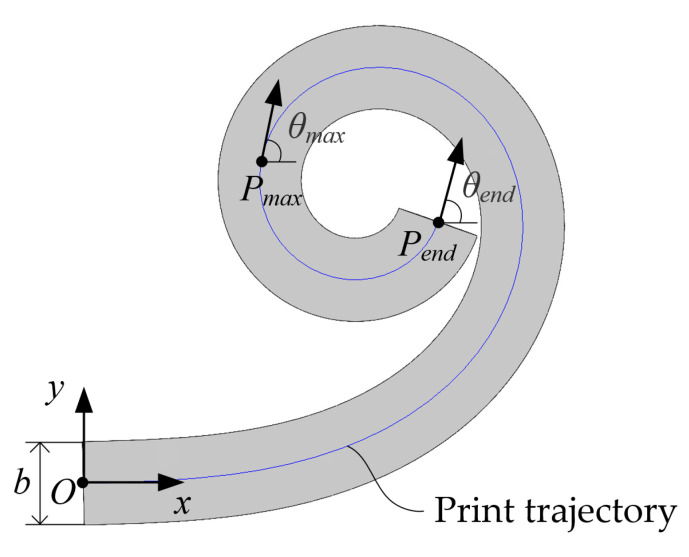
Clothoid printing trajectory.

As shown in Figure 13, this paper designs a clothoid printing trajectory with arc length $s_{end}$ and curvature $k_{end}$ of its endpoint $P_{end}$. It is posited that the curvature at point $P_{\max}$ represents the maximum printable curvature $k_{\max}$ when further printing becomes unfeasible. Furthermore, $s_{\max}$ denotes the arc length measured from the commencement of printing at point *O* to point $P_{\max}$, and *b* is the width of the CFPF after printing.

**Figure 14:**
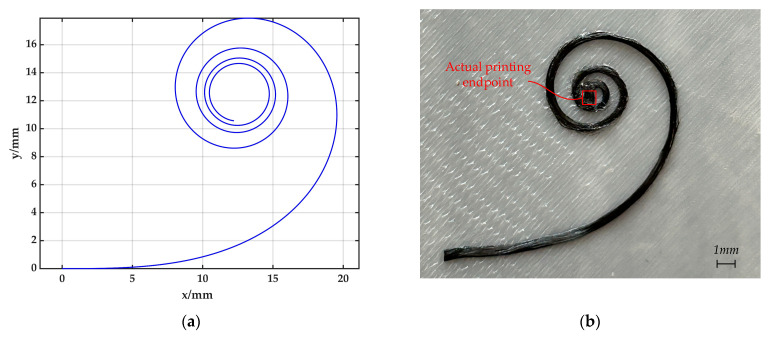
(**a**) Planned clothoid printing trajectory; (**b**) Actual Printing Effect of clothoid printing trajectory.

In order to facilitate accurate measurement and inhibit the folding of CFPF, it is advisable to design the value of $s_{end}$ to be as large as feasible. Given the values $k_{end}=0.5\;{\mathrm{mm}}^{-1}$, $s_{end}=100\;\mathrm{mm}$, and $c_{k}=0.005$, the printing trajectory is illustrated in Figure 14a. By employing the print process parameters as described (printing speed 600 mm/min, printing layer height 0.2 mm, printing temperature 270 °C, ambient temperature 25 °C), the actual printing trajectory, illustrated in Figure 14b, can be achieved.From Figure 14b, it is evident that when $\theta_{\max}=\frac{11}{2}\pi$, the printing process cannot proceed. This issue arises from the clearance, denoted as Δr, existing between the nozzle and the CFPF. When the printing curvature is excessively large, the nozzle tends to revolve around the CFPF, failing to drag the latter and thus, unable to print the intended curvature. At this juncture, Equations (31) and (32) informs us that: s=83.135 mm, $k_{\max}=0.416\;{\mathrm{mm}}^{-1}$.

### 3.3. Simulation and Printing Experiments

**Figure 15:**
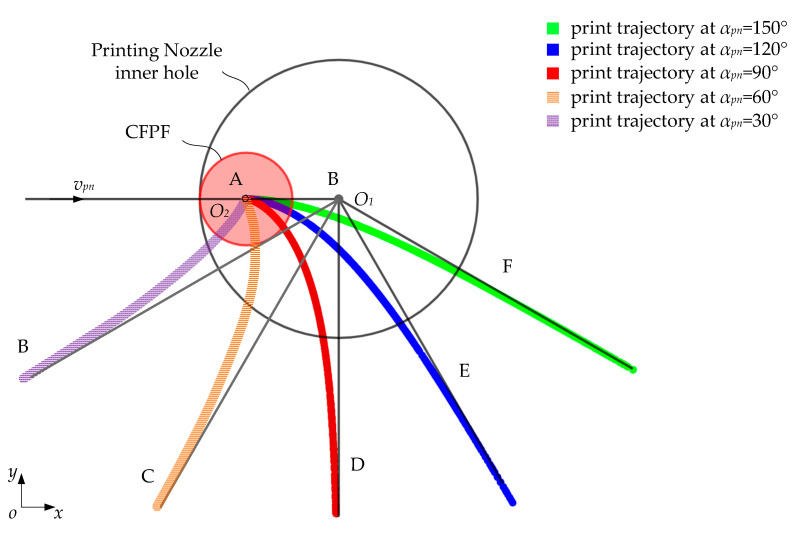
Simulation results of printed trajectory with different corner angles.

To investigate the error patterns and characteristics of printed trajectories at varying corner angles, GeoGebra was employed to simulate these trajectories based on a predefined error model (Δr=0.4 mm). The findings are presented in Figure 15. The simulations show that as the corner angle increases, the line profile decreases monotonically, and the deviation kurtosis first increases and then decreases, while the deviation area ratio first decreases and then increases (Table 2). When the corner angle is 30°, the line profile and the deviation area ratio reach the maximum values of 0.4 mm and 62%, respectively, while the deviation kurtosis has its maximum value of 2.69% when the corner angle is 60°.

To investigate the sensitivity of the compensation method to the corner angle and layer height, trajectories pre- and post-compensation were examined using uniform design experiments (corner angle 30°~150°, layer height 0.1~0.3 mm). The findings are presented in Table 3.

**Figure 16:**
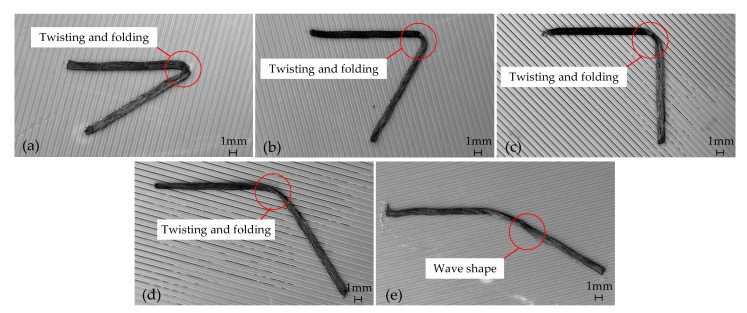
Uncompensated Printing with different corner angles and layer height. (**a**) α*_pn_* = 30°, △*h* = 0.15 mm; (**b**) α*_pn_* = 60°, △*h* = 0.25 mm; (**c**) α*_pn_* = 90°, △*h* = 0.1 mm; (**d**) α*_pn_* = 120°, △*h* = 0.2 mm; (**e**) α*_pn_* = 150°, △*h* = 0.3 mm.

Printing uncompensated trajectory at different corner angles according to the process parameters as described (printing speed 600 mm/min, printing temperature 270 °C, ambient temperature 25 °C, as specified in reference [19]), the results are shown in Figure 16. It is evident that the uncompensated printed trajectory, with a corner of 30°~120°, exhibits significant distortion and folding in the CFPF at the corner. As the angle nears 150°, the distortion and folding effects become less pronounced. However, a wave-like phenomenon emerges, primarily attributed to an excessive layer height (0.3 mm) and insufficient compaction of the CFPF.

**Figure 17:**
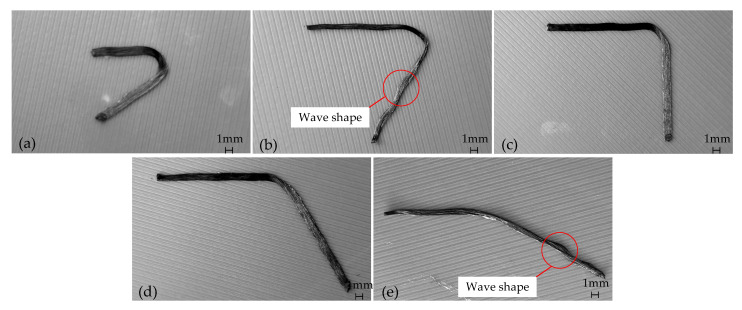
Compensated printing with different corner angles and layer height. (**a**) α*_pn_* = 30°, △*h* = 0.15 mm; (**b**) α*_pn_* = 60°, △*h* = 0.25 mm; (**c**) α*_pn_* = 90°, △*h* = 0.1 mm; (**d**) α*_pn_* = 120°, △*h* = 0.2 mm; (**e**) α*_pn_* = 150°, △*h* = 0.3 mm.

The printed trajectory with different corner angles is compensated by an arc with a curvature of $k_{\max}$, and printed with the process parameters as described above, the result is shown in Figure 17. It is evident that the compensated printing trajectory effectively mitigates the risk of printing defects such as twisting and folding. However, there is a corresponding increase in the geometric error, particularly noticeable when the corner angle is small. Furthermore, when the layer height exceeds 0.25 mm, the printing trajectory exhibits a wave-like phenomenon, as depicted in Figure 17b,e. This is attributed to the insufficient compaction of the CFPF. Consequently, it is suggested that the printing layer height be maintained between approximately 0.1 and 0.2 mm for optimal results.

To analyze the accuracy of the prediction model for the printing trajectory error, we define the prediction deviation of printing trajectory error as follows:

(34) $$\delta =\frac{E_{u}-E_{p}}{E_{u}}\times 100\%$$

where $E_{u}$ is the uncompensated printed trajectory error, and $E_{p}$ is the predicted printed trajectory error. The standard deviation of δ is as follows:

(35) $$\sigma =\sqrt{\frac{1}{n}\sum_{i=1}^{n}{\left(\delta_{i}-\mu \right)}^{2}}$$

where μ is the mean value of δ, n is the total number of samples, and the calculation results are shown in Table 4.

The data in Table 4 demonstrates that with $30{}^{\circ}\leq \alpha_{pn}\leq 150{}^{\circ}$, the error prediction deviation is notably high. This is primarily due to an excessive prediction deviation associated with small corner angles. However, when $90{}^{\circ}\leq \alpha_{pn}\leq 150{}^{\circ}$, the mean prediction deviations for line profile, deviation kurtosis, and deviation area ratio are 36.029%, 47.238%, and 2.045%, respectively. The standard deviations are 3.749%, 1.204%, and 7.674%, while the extreme deviations at 9.176%, 2.875%, and 17.874%. From these results, it is evident that the error model accurately predicts the printing trajectory error for obtuse angle trajectories. Therefore, this model is suitable for forecasting errors in obtuse angle printing trajectories.

Table 3 was consulted to compute the mean, standard deviation, and other pertinent evaluation indices of the printing trajectory error both pre- and post-compensation, the results of which are presented in Table 5.

To assess the correlation between the errors in the printed trajectory (pre- and post-compensation) with respect to the folding angle and layer height, we employ the Pearson correlation coefficient, defined as:

(36) $$Corr\left(A,B\right)=\frac{Cov\left(A,B\right)}{\sqrt{Var\left(A\right)\cdot Var\left(B\right)}}$$

where *A* and *B* denote two random variables, *Cov*(*A*, *B*) signifies the covariance between *A* and *B*, *Var*(*A*) represents the variance of *A*, and *Var*(*B*) represents the variance of *B*.

According to the calculations in Table 3, the correlation coefficients of the printing trajectory error with corner angle before compensation are −0.944, −0.945, and −0.952, while after compensation, they are −0.928, −0.929, and −0.966. The correlation coefficients of the printing trajectory error with layer height before compensation are −0.191, −0.21, and −0.361, whereas after compensation, they are −0.162, −0.149, and −0.391. It is evident that there is a significant negative correlation between the printing trajectory error with corner angle, both pre- and post-compensation, as the correlation coefficients are close to −1. However, the correlation coefficients of the printing trajectory error with layer height, both before and after compensation, are closer to 0, implying a weak correlation. This suggests that the impact of layer height on the printing trajectory error is negligible.

**Figure 18:**
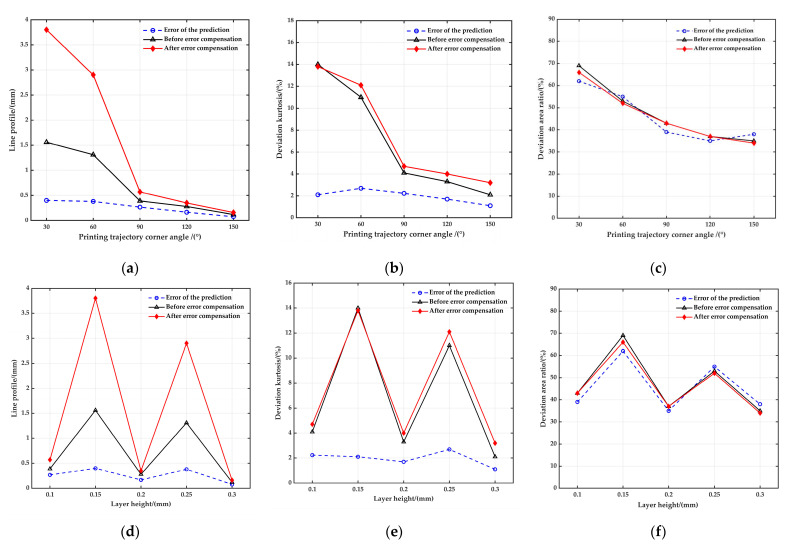
Comparison of error compensation before and after printing trajectories with different corner angle and layer height. (**a**) Line profile versus corner angle; (**b**) deviation kurtosis versus corner angle; (**c**) deviation area ratio versus corner angle; (**d**) line profile versus layer height; (**e**) deviation kurtosis versus layer height; (**f**) deviation area ratio versus layer height.

We plotted the change curve of printing trajectory error with corner angle and layer height as the abscissa, as shown in Figure 18. Figure 18a illustrates that the line profile of a compensated printed trajectory becomes larger when the corner angle is an acute angle. The smaller the corner angle, the more significant the increase in the line profile after compensation. For instance, at a corner angle of 30°, the line profile expands by approximately 2.24 mm. However, if the corner angle is an obtuse angle, the change in the line profile post-compensation is so minor that it can be disregarded. Figure 18b,c illustrate that the deviation kurtosis and deviation area ratio of the printed trajectory remains largely consistent before and after compensation. After compensation, the mean deviation area ratio exhibited a reduction from 47.4% to 31.898%, while the mean deviation kurtosis rose from 6.9% to 7.56%. Additionally, all standard deviations experienced marginal decreases.

In summary, this error compensation technique effectively mitigates issues related to twisting and folding while maintaining high printing accuracy.

## 4. Conclusions

This paper addresses defects such as fiber bundle twisting, folding, and breakage caused by printing trajectory errors in CFRTPCs 3DP. A follow-up theory assumption is proposed to elucidate the formation mechanism of these printing trajectory errors from a theoretical standpoint. It also examines the impact of key geometric parameters, including trajectory curvature, printing nozzle inner diameter, and fiber bundle diameter, on these printing trajectory errors. Moreover, a model for printing trajectory error is established, and a trajectory error compensation method based on maximum printable curvature is proposed. The efficacy of this method is confirmed through error compensation experiments.

The conclusions drawn from the practical printing test and subsequent data analysis are as follows:

1. The error model provides accurate predictions of the printed trajectory error, particularly when the printed trajectory forms an obtuse angle. The average prediction deviations for line profile, deviation kurtosis, and deviation area ratio are 36.029%, 47.238%, and 2.045%, respectively.
2. The layer height demonstrates a low sensitivity to printing trajectory errors. However, the emergence of wave-shaped defects may be observed when the layer height exceeds 0.25 mm. Consequently, it is suggested that the optimal layer height be maintained within the range of 0.1 to 0.2 mm.
3. The correlation between the printed trajectory errors observed before and after compensation and the corner angle is markedly negative. A sharper corner in the printed trajectory corresponds to a larger trajectory error.
4. After compensation, both fiber bundle twisting and folding defects were effectively mitigated. The deviation area ratio decreased by an average of 15.502%, while there was an observed increase in the line profile by an average of 0.824 mm. Additionally, the deviation kurtosis experienced an average increase of 0.66%.

In summary, this trajectory error compensation method effectively mitigates the issues of twisting and folding during printing, while maintaining minimal impact on printing accuracy. These findings suggest that this method significantly improves the defects associated with the CFRTPCs 3DP printing process and could potentially be applied to other continuous fiber printing types.

## Figures and Tables

**Table 1:** PAHT and CFPF material parameters.

PAHT		CFPF	
Parameters	Value	Parameters	Value
Filament diameter, mm	1.75	Filament diameter, mm	0.4
Density, g/cm^3^	1.21	Tensile strength, MPa	1200
Tensile strength, MPa	69.29 ± 1.17	Printing speed, mm/min	300~1000
Bending strength, MPa	112.64 ± 1.6	Printing temperature, °C	260~300
Printing temperature, °C	280~320	Fiber Type	1.5 k
Base plate temperature, °C	80~100	prepreg resin	PAHT

**Table 2:** Error prediction result based on the printing trajectory error model.

Corner Angle	Error Prediction		
	$\mathrm{Line}\;\mathrm{Profile}\;\varsigma_{p}$ /(mm)	$\mathrm{Deviation}\;\mathrm{Kurtosis}\;\chi_{p}$	$\mathrm{Deviation}\;\mathrm{Area}\;\mathrm{Ratio}\;\gamma_{p}$
30°	0.4	2.1%	62%
60°	0.38	2.69%	55%
90°	0.267	2.23%	39%
120°	0.166	1.7%	35%
150°	0.077	1.1%	38%

**Table 3:** Uniform design experiment and results.

No.	Influencing Factors		Uncompensated			Compensated		
	$\mathrm{Corner}\;\mathrm{Angle}\;\alpha_{pn}$ /(°)	Layer Height Δh /(mm)	$\mathrm{Line}\;\mathrm{Profile}\;\varsigma_{u}$ /(mm)	$\mathrm{Deviation}\;\mathrm{Kurtosis}\;\chi_{u}$	$\mathrm{Deviation}\;\mathrm{Area}\;\mathrm{Ratio}\;\gamma_{u}$	$\mathrm{Line}\;\mathrm{Profile}\;\varsigma_{u}$ /(mm)	$\mathrm{Deviation}\;\mathrm{Kurtosis}\;\chi_{u}$	$\mathrm{Deviation}\;\mathrm{Area}\;\mathrm{Ratio}\;\gamma_{u}$
1	30	0.15	1.56	14%	69%	3.8	13.8%	66%
2	60	0.25	1.31	11%	53%	2.9	12.1%	52%
3	90	0.1	0.39	4.1%	43%	0.57	4.7%	43%
4	120	0.2	0.28	3.3%	37%	0.35	4%	37%
5	150	0.3	0.12	2.1%	35%	0.16	3.2%	34%

**Table 4:** Analysis of the fluctuation of error prediction.

Evaluation Index	$30{}^{\circ}\leq \alpha_{pn}\leq 150{}^{\circ}$			$90{}^{\circ}\leq \alpha_{pn}\leq 150{}^{\circ}$		
	$\mathrm{Line}\;\mathrm{Profile}\;\mathrm{Prediction}\;\mathrm{Deviation}\;\delta_{\varsigma }$	$\mathrm{Deviation}\;\mathrm{Kurtosis}\;\mathrm{Prediction}\;\mathrm{Deviation}\;\delta_{\chi }$	$\mathrm{Deviation}\;\mathrm{Area}\;\mathrm{Ratio}\;\mathrm{Prediction}\;\mathrm{Deviation}\;\delta_{\gamma }$	$\mathrm{Line}\;\mathrm{Profile}\;\mathrm{Prediction}\;\mathrm{Deviation}\;\delta_{\varsigma }$	$\mathrm{Deviation}\;\mathrm{Kurtosis}\;\mathrm{Prediction}\;\mathrm{Deviation}\;\delta_{\chi }$	$\mathrm{Deviation}\;\mathrm{Area}\;\mathrm{Ratio}\;\mathrm{Prediction}\;\mathrm{Deviation}\;\delta_{\gamma }$
Mean value	50.687%	60.452%	2.502%	36.029%	47.238%	2.045%
Standard deviation	18.218%	16.484%	7.417%	3.749%	1.204%	7.674%
Max value	74.359%	85%	10.145%	40.714%	48.485%	9.302%
Min value	31.538%	45.61%	−8.571%	31.538%	45.61%	−8.571%
Extreme deviations	42.821%	39.39%	18.716%	9.176%	2.875%	17.874%

**Table 5:** Analysis of the fluctuation of error before and after compensation.

Evaluation Index	Uncompensated			Compensated		
	$\mathrm{Line}\;\mathrm{Profile}\;\varsigma_{u}$ /(mm)	$\mathrm{Deviation}\;\mathrm{Kurtosis}\;\chi_{u}$	$\mathrm{Deviation}\;\mathrm{Area}\;\mathrm{Ratio}\;\gamma_{u}$	$\mathrm{Line}\;\mathrm{Profile}\;\varsigma_{c}$ /(mm)	$\mathrm{Deviation}\;\mathrm{Kurtosis}\;\chi_{c}$	$\mathrm{Deviation}\;\mathrm{Area}\;\mathrm{Ratio}\;\gamma_{c}$
Mean value	0.732	6.9%	47.4%	1.556	7.56%	31.898%
Standard deviation	0.586	4.713%	12.484%	1.498	4.459%	11.569%
Max value	1.56	14%	69%	3.8	13.8%	66%
Min value	0.12	2.1%	35%	0.16	3.2%	34%
Extreme deviations	1.44	11.9%	34%	3.64	10.6%	32%

## Data Availability

The original contributions presented in this study are included in the article. Further inquiries can be directed to the corresponding authors.
